# Correlations of Internet Addiction Severity With Reinforcement Sensitivity and Frustration Intolerance in Adolescents With Attention-Deficit/Hyperactivity Disorder: The Moderating Effect of Medications

**DOI:** 10.3389/fpsyt.2019.00268

**Published:** 2019-04-26

**Authors:** Wei-Hsin Lu, Wen-Jiun Chou, Ray C. Hsiao, Huei-Fan Hu, Cheng-Fang Yen

**Affiliations:** ^1^Department of Psychiatry, Ditmanson Medical Foundation Chia-Yi Christian Hospital, Chia-Yi City, Taiwan; ^2^Department of Child and Adolescent Psychiatry, Chang Gung Memorial Hospital, Kaohsiung Medical Center and College of Medicine, Chang Gung University, Kaohsiung, Taiwan; ^3^Department of Psychiatry and Behavioral Sciences, University of Washington School of Medicine, Seattle, WA, United States; ^4^Department of Psychiatry, Children’s Hospital and Regional Medical Center, Seattle, WA, United States; ^5^Department of Psychiatry, Tainan Municipal Hospital (Managed by Show Chwan Medical Care Corporation), Tainan, Taiwan; ^6^Department of Psychiatry, Kaohsiung Medical University Hospital, Kaohsiung, Taiwan; ^7^Department of Psychiatry, School of Medicine, and Graduate Institute of Medicine, College of Medicine, Kaohsiung Medical University, Kaohsiung, Taiwan

**Keywords:** adolescent, attention-deficit/hyperactivity disorder, internet addiction, reinforcement sensitivity, behavior approach system, behavioral inhibition system, frustration intolerance

## Abstract

**Background:** Deviations in reinforcement sensitivity and frustration-related reactions have been proposed as components of the biopsychosocial mechanisms, which explained the high vulnerability to internet addiction (IA) among individuals with attention-deficit/hyperactivity disorder (ADHD). There is currently limited knowledge on the relationship of IA symptoms with reinforcement sensitivity and frustration intolerance, as well as factors moderating those correlations in this population.

**Objective:** The aims of this study were (1) to examine the associations of IA symptoms severity with reinforcement sensitivity and frustration intolerance and (2) identify the moderators of these associations among adolescents diagnosed with ADHD in Taiwan.

**Methods:** A total of 300 adolescents aged between 11 and 18 years who had been diagnosed with ADHD participated in this study. Their levels of IA severity, reinforcement sensitivity, and frustration intolerance were assessed using the Chen Internet Addiction Scale, behavioral inhibition system (BIS) and behavioral approach system (BAS), and Frustration Discomfort Scale, respectively. The associations of IA severity with reinforcement sensitivity and frustration intolerance were examined using multiple regression analysis. Possible moderators, including medications for ADHD, were tested using the standard criteria.

**Results:** Higher fun seeking on the BAS (*p* = .003) and higher frustration intolerance (*p* = .003) were associated with more severe IA symptoms. Receiving medication for treating ADHD moderated the association between fun seeking on the BAS and severity of IA symptoms.

**Conclusion:** Fun seeking on the BAS and frustration intolerance should be considered as targets in prevention and intervention programs for IA among adolescents with ADHD.

## Introduction

The negative effects of internet addiction (IA) have become a concern in the past decades. IA is characterized by persistent internet use despite negative consequences, loss of control, preoccupation with internet use, increasing amounts of time spent online, and withdrawal symptoms (1). Internet gaming disorders are listed in the “Conditions for Further Study” section in the Fifth Edition of Diagnostic and Statistical Manual of Mental Disorders (DSM-5) (2). Adolescents were raised in an era in which the internet rapidly expanded its influence in daily life.

Attention-deficit/hyperactivity disorder (ADHD) is the most common comorbidity among adolescents referred for treatment of IA (3). Relevant studies have consistently reported associations between IA and ADHD. One study reported that 14% of adults with IA have also been diagnosed with ADHD (4). Individuals with IA have a 2.5 times higher risk of being diagnosed with ADHD according to a meta-analysis (5). Ko et al. (6) discovered that during a 2-year follow-up period, adolescents with significant ADHD symptoms were more likely to develop IA than were those without. Moreover, ADHD symptoms, including inattention and impulsivity/hyperactivity, were more severe in individuals with IA than in healthy controls (5). Evidence suggests that the relationship between ADHD and IA is likely to be bidirectional and mutually interactive. For example, although a 3-year follow-up study reported that children and adolescents with more severe attention problems spent more time playing video games during follow-up (7), a 2-year prospective study discovered that heavy digital media users without ADHD symptoms at baseline had a higher risk of developing ADHD symptoms during the follow-up period (8).

Ko et al. (9) proposed possible biopsychosocial mechanisms to explain the high correlation between ADHD and IA, including avoidance of boredom and delayed reward, striatal dopamine release, compensation for real-life frustrations, impaired inhibition, and deviation in reinforcement sensitivity. Reinforcement sensitivity and frustration may play important roles among these mechanisms. Firstly, patients with ADHD were reported to have deviations in responses to reinforcements, such as rapid habituation to repeated rewards and decreased responses to punishments, which may predispose these individuals to IA because internet activities often provide quick rewards and responses (10). Secondly, adolescents with ADHD often encounter various frustrations in their daily life because of their symptoms. Striatal dopamine release during video gaming (11) may enhance the performance of the game players, thereby helping adolescents with ADHD to compensate for real-life frustrations. In addition, impulsivity, inattention and hyperactivity usually produce frustrations in an interpersonal relationship; therefore, individuals with ADHD may rely more on the internet because it is easier to establish interpersonal relationships online than that in real world. In this view, IA may be a consequence of poor tolerance to frustrations. Recognizing these possible factors which contribute to the strong association between ADHD and IA is crucial to prevention and providing interventions for IA in adolescents with ADHD. However, previous reseaches supporting these proposed mechanisms are still limited. To the best of our knowledge, only one study examined the predictors of IA symptoms in adolescents clinically diagnosed with ADHD (12). Hence, in this study, we focused on the roles of reinforcement sensitivity and frustration intolerance to address these knowledge gaps.

Reinforcement Sensitivity Theory (RST) was developed by Gray and consists of the behavioral inhibition system (BIS) and behavioral approach system (BAS), which are used to identify an individual’s sensitivity to punishment and reward, respectively (13). BAS and BIS can provide explanations for impulsivity and anxiety, respectively (14). Although Gray revised his theory in 2000, making some adjustments to account for the complexity of the constitution and interaction of RST systems (15), many prominent studies have utilized the older RST model (14). Most research on the role of RST in IA has also used the older RST model (12, 16–20). To maintain consistent methodology, we also used the original RST model in this study. Cross-sectional and prospective research on adolescents and adults has identified associations between reinforcement sensitivity and IA symptoms. Specifically, high BAS fun seeking and high BIS have been demonstrated to be positively correlated with the severity of IA in cross-sectional studies (17, 21). A 1-year follow-up study revealed that individuals with higher total BAS and BAS fun seeking were more likely to develop IA (18).

Internet activities are usually characterized by immediate responses and rapid rewards; therefore, deviations in sensitivity to reinforcement may contribute to vulnerability to IA in patients with ADHD (9). Abnormal reinforcement sensitivity is considered a fundamental characteristic of ADHD (10, 22, 23). Research has indicated that patients with ADHD have higher reward sensitivity to immediate reinforcements (24), more rapid habituation to repeated reinforcements (25), and lower response to punishment (25, 26). Impulsivity, a prominent symptom of ADHD, is commonly reported in individuals with IA (19, 27), and it has been linked to BAS functioning (28). Studies on subjects with ADHD have also reported that higher BAS fun seeking, BAS drive, and BIS are positively associated with IA symptoms (12, 19). However, few studies have been conducted on patients clinically diagnosed with ADHD, and more information is required to support the role of reinforcement sensitivity in patients with ADHD. Moreover, evidence suggests that the effects of reinforcement sensitivity vary under different conditions. Research has found that increased age and low parental occupational SES were significantly associated with severe internet addiction symptoms in adolescents with ADHD (12). Family factors have been reported to moderate the association between reinforcement sensitivity and behavioral problems in children and adolescents (29). Adolescents receiving medication for ADHD exhibited problematic online gaming symptoms and concurrent decreases in BAS and BIS scores (20). Moreover, reinforcement sensitivity was reported to be a vulnerable factor of psychiatric disorders, such as depression, anxiety, and substance abuse (30). However, no study has explored the moderating effects of socio-demographic characteristics, medical treatment for adolescents with ADHD, and concurrent psychiatric disorders on the association between IA symptoms and reinforcement sensitivity in adolescents with ADHD.

Frustration intolerance refers to the difficulty accepting that reality does not correspond to personal desires (31). It is a type of irrational belief related to emotional and behavioral problems based on the theory of rational emotive behavior therapy (32). Adolescents with IA have been reported to have higher frustration intolerance that healthy controls (21), indicating that frustration intolerance is associated with difficulty with self-control (33). Aversion to delayed reward, which may be a source of frustration, is a core feature of ADHD (22). Researchers have observed high frustration intolerance in youths with ADHD (34–36). Hypothesizing that frustration intolerance is a predictor of IA symptoms in individuals with ADHD is therefore reasonable. Nevertheless, no study has examined the relationship between frustration intolerance and IA symptoms in adolescents with ADHD. Considering the high risk of IA in adolescents with ADHD, understanding the role of frustration intolerance in predicting IA may facilitate the design of effective cognitive behavioral therapies targeting ADHD adolescents with IA. Furthermore, sex is currently the only factor that has been proven to moderate the correlation between frustration intolerance and IA in adolescents (21). In the present study, we investigated whether socio-demographic characteristics, medical treatment for adolescents with ADHD, and concurrent psychiatric disorders moderate this relationship between frustration intolerance and IA symptoms in adolescents with ADHD.

The aims of the present study were to examine the correlations between IA severity and reinforcement sensitivity and frustration intolerance as well as identify moderators of these correlations in adolescents in Taiwan who have been diagnosed with ADHD. We hypothesized that both reinforcement sensitivity and frustration intolerance exhibit significant correlations with IA severity, and that these correlations may be moderated by sociodemographic characteristics, ADHD symptoms and treatment, psychiatric comorbidities, and parental factors.

## Materials and Methods

### Participants

Participants for this study were recruited from the child and adolescent psychiatric outpatient clinics of two medical centers in Kaohsiung, Taiwan. Adolescents aged between 11 and 18 years, who visited the outpatient clinics and have been diagnosed with ADHD according to the diagnostic criteria specified in the DSM-5 (2), were consecutively invited to participate in this study during the period from August 2013 to July 2015. ADHD was diagnosed on the basis of multiple data sources, including (i) an interview with a child psychiatrist; (ii) clinical observation of the participant’s behavior; and (iii) medical history provided by parents and parent-reported severity of ADHD symptoms assessed from the short version of Swanson, Nolan, and Pelham, Version IV Scale (SNAP-IV)-Chinese version (37, 38). Adolescents with intellectual disabilities, schizophrenia, bipolar disorder, autistic disorder, communication difficulties, or cognitive deficits that adversely affect their ability to understand the study purpose or complete the questionnaires were excluded. A total of 333 adolescents who had been diagnosed with ADHD and their parents were selected for this study, 300 of which (90.0%) agreed to participate in this study and were interviewed by research assistants through a questionnaire. Of the 33 adolescents who refused to join this study, 19 refused because of their parents’ opinions and 14 refused because of their own opinions. The Institutional Review Boards of Kaohsiung Medical University and Chang Gung Memorial Hospital, Kaohsiung Medical Center, approved the study. Written informed consent was obtained from all participants before assessment.

### Measures

*Internet addiction.* We used the Chen Internet Addiction Scale (CIAS) to assess participant self-reported severity of IA symptoms in the recent 1 month. The CIAS contains 26 items evaluated on a 4-point Likert scale, with scores ranging from 26 to 104 (39); a higher total score indicates more severe IA symptoms. The CIAS has been commonly used to assess internet addiction among children and adolescents in Taiwan (1, 40). The internal reliability (Cronbach’s α) of the CIAS was .94 in the present study.

*Reinforcement sensitivity.* The Chinese version of BIS and BAS scales contain 20 items evaluated on a 4-point Likert scale; these scales assess participants’ self-reported sensitivity for the two motivational systems according to RST (13, 28, 41). The BIS measures the degree to which respondents expect to feel anxiety when confronted with cues for punishment. The BAS includes subscales of reward responsiveness, drive, and fun seeking, which measure the degree to which rewards lead to positive emotions, an individual’s tendency to actively pursue goals, and the tendency to seek out and impulsively engage in potentially rewarding activities, respectively. A higher total score on the subscale indicates a higher level of reinforcement sensitivity. The Chinese versions of BIS and BAS scales were translated from the original version using the standard forward-, backward-, and pretest-step method and have been reported to have good criterion and construct validity in the previous study on Taiwanese population (41). The BIS and BAS scales have been used to assess reinforcement sensitivity among adolescents in Taiwan (12). The Cronbach’s α of the four subscales ranged from .68 to .83 in the present study.

*Frustration intolerance.* In the present study, the Chinese version of Frustration Discomfort Scale (FDS) was used to evaluate the self-reported frustration intolerance belief of participants (21, 42). The FDS contains 28 items evaluated on a 5-point Likert scale, with scores ranging from 28 to 140; a higher total score indicates higher frustration intolerance beliefs. The Chinese versions of FDS scales was translated from the original version using the standard forward-, backward-, and pretest-step method and have been used to evaluate frustration intolerance beliefs in Taiwanese adolescents (21). The Cronbach’s alpha of the FDS was.90 in the present study.

*ADHD symptoms and treatment.* In the present study, the short version of SNAP-IV-Chinese version was used to assess the parent-reported severity of ADHD symptoms for adolescents in the recent 1 month. This short version of SNAP-IV-Chinese version is a 26-item rating instrument that includes the core Fourth Edition of DSM (DSM-IV)-derived ADHD subscales of inattention, hyperactivity/impulsivity, and symptoms of oppositional defiant disorder with good criterion and construct validity (37, 38). Each item was rated on a 4-point Likert scale from 0 (not at all) to 3 (very much). In this study, the total scores for the inattention and hyperactivity/impulsivity subscales were used for analysis. The Cronbach’s α of these two subscales was .86 and .88, respectively. Whether the participants received medication for ADHD was determined based on parent reports and participant medical records.

*Psychiatric comorbidities.* The depressive disorders, anxiety disorders, tic disorders, and autism spectrum disorders (ASDs) of participants were assessed based on the clinical interviews and chart reviews by three child psychiatrists. Those who had been diagnosed with any ASD and low intelligence (defined as a score less than 70 on the Chinese version of the Fourth Edition of the Wechsler Intelligence Scale for Children [43]) or those who had communication difficulties were not invited to participate in this study. For the purpose of analysis, psychiatric diagnoses were categorized as depressive or anxiety disorders, tic disorders, and ASDs.

*Parental factors.* The present study evaluated the marital status of the parents of participants (married and living together vs. divorced or separated) and assessed their occupational socioeconomic status (SES) using Close-Ended Questionnaire of the Occupational Survey (CEQ-OS) (44). Parents choose their occupations from 14 categories in the CEQ-OS, which were further classified into five levels according to their occupational socioeconomic status. A higher level indicates higher occupational socioeconomic status. The CEQ-OS has been proven to possess excellent reliability and validity and has been commonly used in studies on children and adolescents in Taiwan (44). In the present study, levels I, II, and III of the CEQ-OS were classified as low occupational SES, whereas levels IV and V were classified as high occupational SES. This questionnaire was completed by parents.

### Procedure

The research assistants conducted interviews using the CIAS, BIS/BAS, and FDS to collect data from adolescents. Their parents completed SNAP-IV under the direction of the research assistants. Data analysis was performed using SPSS 20.0 statistical software (SPSS Inc., Chicago, IL, USA).

### Statistical Analysis

Because that there were several factors examined in this study, we used two-step statistical analyses to examine the correlation of IA severity with reinforcement sensitivity and frustration intolerance and reduced the possibility of multiple comparison. In the first step, we used Pearson’s correlation and *t* test to select possible factors predicting IA severity for further analysis, including sociodemographic characteristics, ADHD symptoms and treatment, psychiatric comorbidities, parental factors, reinforcement sensitivity, and frustration intolerance. The significant factors in the first step were used in the second step, which consisted of a multiple regression analysis that was used to evaluate the correlations of reinforcement sensitivity and frustration intolerance with IA severity by controlling for the effects of other factors. A two-tailed *p* value of less than 0.05 was considered statistically significant.

We also used standard criteria (45) to examine whether the associations of reinforcement sensitivity and frustration intolerance with IA severity differed in terms of sociodemographic characteristics, ADHD symptoms and treatment, psychiatric comorbidities, or parental factors. According to the criteria, moderation occurred when the interaction term for the predictor (reinforcement sensitivity and frustration intolerance) and the hypothesized moderator were significantly associated with the dependent variable (IA severity) in multiple regression analysis after controlling for the main effects of both the predictors and hypothesized moderator variables. In this study, if reinforcement sensitivity, frustration intolerance, and hypothesized moderators were significantly associated with IA symptoms, then the interactions (reinforcement sensitivity or frustration intolerance × hypothesized moderators) were further selected for multiple regression analysis to examine the moderating effects.

## Results

### Sociodemographic Characteristics and Correlates of IA Symptoms

**Figure 1:**
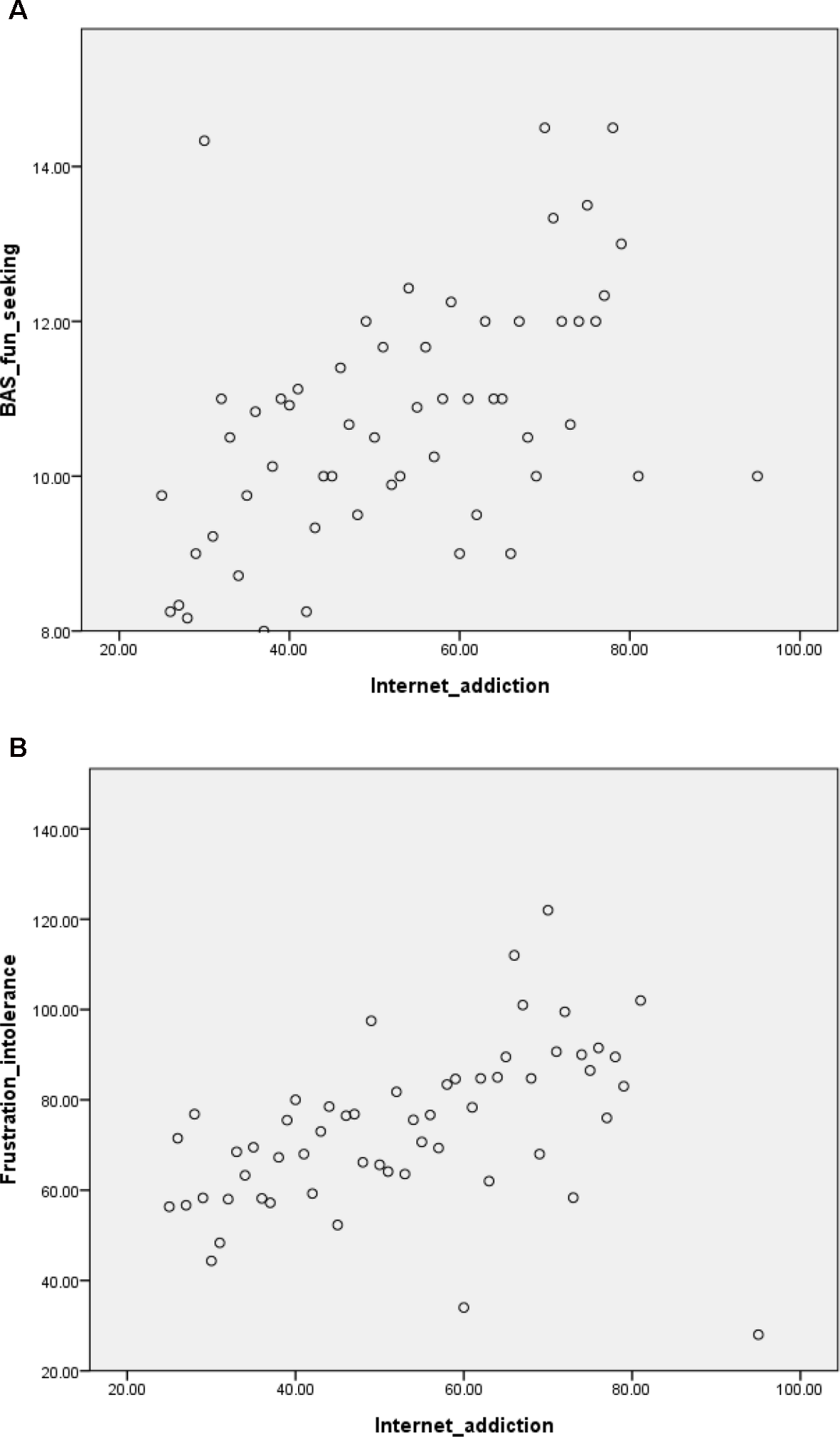
Scatter plots of correlations between internet addiction symptoms and fun seeking on the Behavioral Approach System (BAS) scale **(Figure 1A)** and between internet addiction symptoms and frustration intolerance **(Figure 1B)**.

**Table 1** presents the sociodemographic and ADHD characteristics, comorbidities, IA severity, and BAS/BIS and FDS scores of participants. **Table 2** lists the correlations of IA severity with age, ADHD symptoms, BIS/BAS and FDS scores, as examined using Pearson’s correlation. According to Cohen (46), older age, more severe inattention and oppositional symptoms, higher score for fun seeking on the BAS, and higher frustration intolerance belief on the FDS were weakly but significantly correlated with more severe IA symptoms. **Figure 1** shows the scatter plots of the correlations between IA symptoms and fun seeking on the BAS and between IA symptoms and FDS score.

**Table 1:** Sociodemographic and ADHD characteristics, comorbidities, internet addiction severity, and levels of BAS/BIS and FDS (N = 300).

	*n* (%)	Mean (SD)	Range
Age (years)		12.8 (1.8)	10–18
Sex			
Girls	41 (13.7)		
Boys	259 (86.3)		
Education (years)		7.0 (1.8)	4–12
Parental marriage status			
Married and live together	231 (77.0)		
Divorced or separated	69 (23.0)		
Paternal occupational socioeconomic status			
High	125 (41.7)		
Low	175 (58.3)		
Maternal occupational socioeconomic status			
High	94 (31.3)		
Low	206 (68.7)		
ADHD symptoms on the SNAP-IV			
Inattention		12.7 (5.8)	0–27
Hyperactivity/impulsivity		8.8 (6.0)	0–27
Oppositional		9.8 (5.7)	0–24
Receiving medication for ADHD	254 (84.7)		
Comorbidity			
Depressive or anxiety disorders	40 (13.3)		
Tic disorders	34 (11.3)		
Autism spectrum disorders	34 (11.3)		
Severity of Internet addiction on the CIAS		47.7 (14.1)	25–95
BIS/BAS			
BIS		19.3 (3.7)	8–28
Reward responsiveness on the BAS		16.2 (3.3)	5–20
Drive on the BAS		12.2 (2.9)	4–16
Fun seeking on the BAS		10.6 (2.7)	4–16
FDS		71.4 (25.4)	28–135

ADHD, attention-deficit/hyperactivity disorder; BAS, behavior approach system; BIS, behavior inhibition system; CIAS, Chen Internet Addiction Scale; FDS, Frustration Discomfort Scale; SNAP-IV, Swanson, Nolan, and Pelham, Version IV Scale.

**Table 2:** Correlation of age, ADHD symptoms, BIS/BAS, and FDS with internet addiction severity: Pearson’s correlation.

	Internet addiction severity Pearson’s *r*	*p*
Age (years)	.142	.014
ADHD symptoms on the SNAP-IV		
Inattention	.145	.012
Hyperactivity/impulsivity	.085	.142
Oppositional	.170	.003
BIS/BAS		
BIS	.106	.066
Reward responsiveness on the BAS	.004	.943
Drive on the BAS	.048	.403
Fun seeking on the BAS	.261	<.001
FDS	.290	<.001

ADHD, Attention-deficit/hyperactivity disorder; BAS, Behavior Approach System; BIS, Behavior Inhibition System; FDS, Frustration Discomfort Scale; SNAP-IV, Swanson, Nolan, and Pelham, Version IV Scale.

**Table 3** presents the differences in IA severity between participants with various sociodemographic characteristics, medication status, and psychiatric comorbidities. The results indicated that adolescents with low paternal and maternal occupational SES exhibited more severe IA symptoms than did those with high paternal and maternal occupational SES. Adolescents receiving medication for ADHD had less severe IA symptoms than did those not receiving medication for ADHD.

**Table 3:** Comparison of internet addiction severity of participants according to sociodemographic characteristics, ADHD characteristics, and comorbidities.

	Internet addiction severity Mean (SD)	*t*	*P*
Sex			
Girls (*n* = 41)	49.2 (16.2)	.715	.475
Boys (*n* = 259)	47.5 (13.8)		
Parental marriage status:			
Intact (*n* = 231)	47.8 (14.5)	.151	.880
Broken (*n* = 69)	47.5 (13.1)		
Paternal occupational SES			
High (*n* = 125)	45.7 (12.7)	−2.108	.036
Low (*n* = 175)	49.1 (14.9)		
Maternal occupational SES			
High (*n* = 94)	44.4 (12.0)	−2.734	.007
Low (*n* = 206)	49.2 (14.8)		
Receiving medication for ADHD			
No (*n* = 46)	53.1 (13.4)	2.830	.005
Yes (*n* = 254)	46.7 (14.1)		
Comorbidity			
Depressive or anxiety disorders			
No (*n* = 260)	47.8 (13.9)	.254	.800
Yes (*n* = 40)	47.2 (15.6)		
Tic disorders			
No (*n* = 266)	47.7 (14.3)	.115	.909
Yes (*n* = 34)	47.4 (12.9)		
Autism spectrum disorders			
No (*n* = 266)	47.7 (14.3)	−.027	.979
Yes (*n* = 34)	47.8 (13.0)		

ADHD, Attention-deficit/hyperactivity disorder; SES, socioeconomic status; SNAP-IV, Swanson, Nolan, and Pelham, Version IV Scale.

### Testing of Moderators

As described in the Statistical analysis section, significant factors in the first step were selected for further multiple regression analysis in the second step to detect the independent factors related to IA symptoms (Model I in **Table 4**). The results indicated that low maternal occupational SES, higher fun seeking on the BAS, and higher frustration intolerance belief on the FDS were associated with more severe IA symptoms, whereas receiving medication for ADHD was associated with less severe IA.

**Table 4:** Associated factors and moderators of internet addiction severity.

	Model I			Model II		
	β	*t*	*p*	β	*t*	*p*
Age	.067	1.199	.232	.071	1.262	.208
Low paternal occupational SES	.110	1.940	.053	.119	2.121	.035
Low maternal occupational SES	.125	2.226	.027	−.358	−1.470	.143
Inattention symptoms on the SANP-IV	.038	.580	.563	.039	.603	.547
Oppositional symptoms on the SANP-IV	.077	1.183	.238	.061	.949	.343
Receiving medication for ADHD	−.113	−2.061	.040	−.077	−.312	.755
Fun seeking on the BAS	.175	2.948	.003	.300	1.582	.115
FDS	.180	3.048	.003	−.206	−1.336	.183
Low maternal occupational SES x Fun seeking on the BAS				.051	.200	.842
Receiving medication for ADHD x Fun seeking on the BAS				.511	2.463	.014
Low maternal occupational SES x FDS				−.298	−1.009	.314
Receiving medication for ADHD x FDS				.244	1.310	.191
F	7.827			6.151		
*p*	<.001			<.001		
Adjusted R^2^	.154			.171		

ADHD, Attention-deficit/hyperactivity disorder; BAS, Behavior Approach System; FDS, Frustration Discomfort Scale; SES, socioeconomic status; SNAP-IV, Swanson, Nolan, and Pelham, Version IV Scale.

Because maternal occupational SES and receiving medication for ADHD were significantly associated with IA symptoms, the interactions among the predictors (reinforcement sensitivity and frustration intolerance) and possible moderators (maternal occupational SES and receiving medication for ADHD) were included in the multiple regression analysis based on the standard criteria proposed by Baron and Kenny (45) described in the Statistical analysis section (Model II in **Table 4**). The results indicated that the interaction between fun seeking on the BAS and receiving medication for ADHD was significantly associated with IA severity, suggesting that receiving medication for ADHD moderated the association between fun seeking on the BAS and IA severity. The results of further analysis revealed a significant association between fun seeking on the BAS and IA severity only in participants receiving medication for ADHD (β = .154, *t* = 2.301, *p* = .022) and not in those not receiving medication for ADHD (β = .291, *t* = 2.004, *p* = .052).

## Discussion

The results of this study revealed that although both BAS fun seeking and frustration intolerance were positively associated with IA symptoms, medication treatment for ADHD moderated the relationship between fun seeking on the BAS and IA severity. To the best of our knowledge, this is the first study to identify the moderators of correlations of IA symptoms with reinforcement sensitivity and frustration intolerance in adolescents with ADHD.

BAS fun seeking represents the tendency to seek stimuli and response to proximal rewards (47). Internet use provides individuals with activities having various modes of stimulation and rapid rewards; therefore, individuals with high BAS scores may be more likely to develop IA. A bidirectional relationship remains possible, as indicated in a longitudinal study (18). The present study discovered that the association between BAS fun seeking and IA severity is significant only in adolescents receiving medication for ADHD. This finding is different from the results of other studies, which have concluded significant associations of BAS fun seeking and IA severity in adolescents or young adults (16–18) and adolescents with ADHD (12). The results of our study may indicate that the effects of RST subsystems on IA severity are complex and interactive. Gray’s revised version of the RST includes the subsystems of the BAS, Fight/Flight/Freeze System (FFFS), and BIS (14, 15). BAS controls approach behavior, and FFFS controls avoidance behavior to aversive stimuli. Both BAS and FFFS are activated during an event that includes both rewarding and aversive stimuli, resulting in a motivational conflict. BIS is then activated by the motivational conflict, and the ongoing behavior is inhibited while directing the individual’s attention to the source of conflict (14). Although internet use produces immediate rewards and relief from boredom, it also frequently results in negative consequences that may lead to motivational conflict. Therefore, IA symptoms may be influenced by the result of these mixed interactions of RST subsystems. Moreover, BAS functioning is considered to be based on dopaminergic systems in the CNS (48), which has also been the primary focus of hypotheses on ADHD etiology (49, 50). Deviation in the dopaminergic process may be a mechanism underlying the difference in association between BAS fun seeking and IA in adolescents with ADHD with and without medication. Dopaminergic and noradrenergic neurotransmission are targets of the most commonly used ADHD medications (i.e. methylphenidate and atomoxetine) in Taiwan. One study discovered that 3 months of methylphenidate and atomoxetine treatment in adolescents with ADHD was associated with a decreased score on the BAS scale (20). ADHD medications may modulate the dopaminergic and noradrenergic systems in the brain and thus affect the relationship between BAS fun seeking and IA severity. The association between BAS fun seeking and IA in general adolescents from the general population and those treated with medication for ADHD, but not in ADHD adolescents without medication, may reflect the normalizing effect of ADHD medications on reinforcement sensitivity. This renders the association between BAS fun seeking and IA in adolescents with ADHD who take medication as more similar to that in adolescents from the general population. However, other possible explanations include differences between medication-treated and medication-free groups in terms of baseline demographic or symptom characteristics. The causal relationship of the effects of ADHD medication on the association between BAS fun seeking and IA severity requires further clarification by prospective studies.

In the present study, frustration tolerance was demonstrated to be a significant predictor of IA severity after controlling for other correlates in the regression model. The theory underlying rational emotive behavior therapy proposes that irrational beliefs triggered by events lead to subsequent negative consequences (51). Conversely, Ko et al. suggested that early internet exposure may lead adolescents to become accustomed to environments with immediate gratification, and they may have a limited ability to tolerate frustrations, prompting the development of the irrational belief of frustration intolerance (21). Individuals with ADHD may experience a great deal of frustration in daily life because of deficits in attention and executive function. After thoughts characteristic of frustration intolerance have been provoked, internet activities may serve as coping strategies for tension relief. The results of this study suggest that the frustration intolerance belief requires adequate evaluation and intervention when managing or preventing IA in adolescents with ADHD.

The current study discovered that lower maternal SES was associated with higher IA severity in adolescents with ADHD. Family SES has been proven to play a pivotal role in adolescent health conditions, and parental SES has been demonstrated to influence depression, obesity, and self-rated health among adolescents in the US (52). Children and adolescents from families with higher SES tend to exhibit healthier behaviors (53). Moreover, parenting is crucial to managing ADHD symptoms, and parents with higher SES may be more likely to have access to ADHD-related psychoeducational information. Additionally, parents with higher SES may have more knowledge on appropriate internet use, and therefore, may be more likely to monitor their children. In traditional Taiwanese families, mothers more commonly manage home routines and primarily serve as child caretakers. Therefore, the responsibility of monitoring and controlling internet use may be more commonly taken up by mothers in Taiwan. However, because the concept of gender equality has evolved alongside the increasing prevalence of double-income households in Taiwan, parental influence on IA still warrants careful consideration. One study reported that parental SES predicts IA severity in adolescents with ADHD, but maternal SES does not (12). Overall, evidence supports the phenomenon that parental SES is a critical correlate of IA in adolescents with ADHD.

Established treatment modality for IA is lacking. Medications that have been studied included escitalopram, bupropion, methylphenidate, and atomoxetine (54). Methylphenidate and atomoxetine were reported to be associated with decreased severity of online gaming and BAS/BIS scores in adolescents with ADHD (20). The results of this study support the need for further investigation on the role of ADHD medications in treatment for adolescents with ADHD. Cognitive behavioral therapy is the major non-pharmacological intervention for IA among previous studies (54). Our study indicates that future research may examine the efficacy of incorporating management of frustration intolerance belief and the tendency of fun seeking in cognitive behavioral interventions in treating patients with ADHD and IA. Moreover, the effect of ADHD medications on both fun seeking and IA severity should be monitored during such interventions.

Several limitations of this study require careful consideration. Measurements were all self-reported; therefore, common method bias cannot be completely ruled out. Adding clinical interview in the evaluation process will improve the diagnostic validity in future studies. Psychometrics of the Chinese versions of BIS-BAS scale and FDS used in the adolescent population warrant further examination. The cross-sectional design limited the capability of forming conclusions regarding causality. Participants were recruited from outpatient departments, and individuals with ADHD who were not receiving clinical care were not approached, meaning that the results may not be generalizable to all adolescents with ADHD. Medications for treating ADHD were not specified in our study; therefore, variety in the effects of medications may have introduced bias into the results. However, methylphenidate and atomoxetine are the only two compounds that have been approved for treating ADHD in Taiwan and comprise almost all medications used for treating ADHD (55, 56). Last but not least, the types of internet activities were not reported in this study. There have been debates on whether different problematic online behaviors, such as interent gaming, online social networking and online shopping should be viewed as one single entity or different distinct behaviors driven by various gratifications (57). Whether differences exist among correlations of various internet activities with reinforcement sensitivity and frustration intolerance requires further study. It is also recommended in further research to apply a prospective design, as well as examine the effects of various ADHD medications.

## Conclusion

The results of the current study indicated that BAS fun seeking and frustration intolerance belief were significantly associated with IA severity in adolescents with ADHD. Differences were observed in the association between BAS fun seeking and IA between participants receiving ADHD medication and those not receiving ADHD medication. Reward sensitivity and frustration intolerance require attention during prevention and management programs for IA in adolescents with ADHD. The effect of ADHD medication should also be considered when evaluating the relationship between reinforcement sensitivity and IA.

## Ethics Statement

This study was carried out in accordance with the recommendations of Kaohsiung Medical University with written informed consent from all subjects. All subjects gave written informed consent in accordance with the Declaration of Helsinki. The protocol was approved by the Kaohsiung Medical University.

## Author Contributions

W-HL: conception and design of the study, drafting the manuscript. W-JC: conception and design of the study, drafting the manuscript. RH: drafting the manuscript. H-FH: acquisition and analysis of data. C-FY: conception and design of the study, acquisition and analysis of data, drafting the manuscript or figures.

## Conflict of Interest Statement

The authors declare that the research was conducted in the absence of any commercial or financial relationships that could be construed as a potential conflict of interest.
